# Ternary Dimension-Synergistic Conductive Architecture Enabling High-Rate, Low-Temperature and Extended-Cycling Nickel-Rich NCA Lithium-Ion Batteries

**DOI:** 10.3390/ma19101956

**Published:** 2026-05-09

**Authors:** Zhongyuan Li, Hongda Yang, Minhu Xu, Xiaohua Tian

**Affiliations:** 1 Electric Power Research Institute, State Grid Heilongjiang Electric Power Co., Ltd., Harbin 150030, China; 2 China Energy Consultation (Beijing) Electric Power Research Institute, Beijing100055, China

**Keywords:** low-temperature lithium-ion battery, 18650 cylindrical cell, nickel-rich NCA cathode, graphene nanoplatelet, multi-walled carbon nanotube, ternary conductive additive

## Abstract

The severe performance degradation of lithium-ion batteries at low temperatures limits their applications in extreme environments. Herein, we report the development of a low-temperature-capable 2.5 Ah 18650 cylindrical battery employing a LiNi_0.8_Co_0.15_Al_0.05_O_2_ cathode with optimized conductive additive formulations. The ternary conductive architecture is rationally designed based on dimensional complementarity: a zero-dimensional Super P (SP) nanoparticle ensures percolation through point-to-point contacts, a one-dimensional multi-walled carbon nanotube (MWCNT) establishes long-range electron highways via line-to-point bridging, and a two-dimensional graphene nanoplatelet (GNP) provides face-to-point encapsulation of active particles, mechanically buffering volume expansion and suppressing interfacial degradation. This hierarchical point–line–plane network generates redundant electron transport pathways while steric hindrance effects mitigate aggregation of each component. Through systematic comparative investigation of GNP/MWCNT/SP ternary and MWCNT/SP binary conductive systems, we elucidate the distinct roles of low-dimensional nanocarbons in electrochemical performance enhancement. Film resistivity measurements reveal that the ternary system achieves a 67% reduction in cathode resistivity (to 9.1 Ω·cm at 20 °C) compared to conventional SP (27.5 Ω·cm), outperforming previously reported binary nanocarbon systems for high-nickel cathodes (typically 40–55% reduction at comparable loadings). This enhancement is achieved at a constant total conductive additive loading of 2.5 wt%, demonstrating that dimensional optimization rather than quantity increase governs electrical transport properties. Electrochemical evaluations demonstrate that the fabricated 18650 cells deliver exceptional rate capability (10C continuous and 20C pulse discharge) and remarkable low-temperature performance (76.8% capacity retention at −40 °C under 1C). Notably, while both conductive formulations exhibit comparable rate performan ce and temperature adaptability, the ternary GNP/MWCNT/SP system demonstrates significant superiority in cycling stability, achieving 94.9% capacity retention after 1000 cycles at ambient temperature versus inferior retention for the binary counterpart. Electrochemical impedance spectroscopy analyses indicate reduced polarization and enhanced lithium-ion diffusion kinetics in the ternary system. This study establishes a high-performance low-temperature 18650 battery chemistry and provides quantitative mechanistic insights into how dimensional synergy in conductive additive design governs the rate capability, thermal behavior, and cycling stability of nickel-rich cathodes operating under extreme conditions.

## 1. Introduction

Lithium-ion batteries (LIBs), as the most competitive electrochemical energy storage technology, have been widely deployed in electric vehicles, aerospace systems, and polar exploration equipment. However, LIBs face severe performance degradation under low-temperature conditions (<−20 °C), constraining their reliable applications in high-latitude regions and extreme-climate environments [1,2]. Previous studies have demonstrated that when the temperature drops to −40 °C, battery capacity may plummet to merely 12% of the rated value, with power density declining by over 80% [3]. This performance deterioration primarily originates from the sharply increased electrolyte viscosity, reduced solid-state lithium-ion diffusion coefficients, and sluggish charge-transfer kinetics at low temperatures, leading to intensified electrode polarization and elevated lithium plating risks [4,5].

In recent years, high-nickel ternary cathode materials such as LiNi_x_Co_y_Al_1−x−y_O_2_ (NCA, x ≥ 0.8) have emerged as the mainstream choice for high-energy-density LIBs due to their high specific capacity (>200 mAh/g) and relative cost advantages [6]. Nevertheless, NCA materials confront more formidable challenges under low-temperature operation: on one hand, their intrinsically low electronic conductivity (~10^−9^ S/cm) further amplifies electron transport resistance at reduced temperatures; on the other hand, high-nickel systems are prone to particle microcracking and surface rock-salt phase transformation during cycling, where low-temperature lithium plating coupled with mechanical stress accelerates capacity fade [7,8]. Therefore, constructing efficient and stable electron-conductive networks is crucial for enhancing the performance of NCA-based low-temperature LIBs.

Conductive additives, as critical components of electrodes, directly influence the electronic accessibility of active materials and electrode structural integrity [9]. Conventional zero-dimensional carbon blacks (such as Super P) form conductive networks through point-to-point contacts, yet require high loadings (2–3 wt%) and struggle to accommodate the thick-electrode polarization demands of high-energy-density electrodes [10,11]. One-dimensional multi-walled carbon nanotubes (MWCNTs), leveraging their high aspect ratios (>1000) and exceptional axial conductivity (10^4^–10^5^ S/m), can establish three-dimensional line-to-point contact networks at low loadings (0.2–0.5 wt%), significantly reducing electrode tortuosity and enhancing rate capability [12,13]. However, the rigid tubular structure of MWCNT is susceptible to contact failure induced by volume variations during cycling, and the inter-tube voids are insufficient to fully cover active particle surfaces, resulting in inadequate long-term charge/discharge stability [14].

Two-dimensional graphene nanoplatelets (GNP), as emerging conductive additives, exhibit in-plane ultrahigh conductivity (10^5^ S/m), large specific surface area, and superior mechanical flexibility, suitable for applications as supercapacitor electrodes [15]. GNP can encapsulate active particles through face-to-point contact modes, effectively buffering volume expansion and suppressing particle crack propagation during electrochemical cycling [16]. Nevertheless, the high aspect ratio and strong van der Waals force of GNP render them highly prone to face-to-face restacking aggregation in electrode slurries, which not only diminishes effective conductive area but also obstructs lithium-ion transport channels, leading to deteriorated electrode kinetics [17]. Consequently, single-dimensional conductive additives are incapable of simultaneously satisfying the multifaceted requirements of high-energy-density batteries for conductivity, mechanical stability, and ion transport.

To overcome the limitations of individual conductive additives, multi-dimensional hybrid conductive systems have attracted extensive attention in recent years. Theoretical studies indicate that synergistic combinations of zero-dimensional carbon black, one-dimensional carbon nanotubes, and two-dimensional graphene can construct hierarchical “point–line–plane” conductive networks, where steric hindrance effects suppress aggregation of each component while forming redundant electron transport pathways [18]. Specifically, the linear structure of MWCNT can intercalate between GNP layers, physically isolating and preventing their restacking; simultaneously, the two-dimensional flexible surface of GNP can fill inter-tube voids of MWCNT, enhancing contact area with active particles. This synergistic effect is anticipated to concurrently improve electrode electronic conductivity, mechanical resilience, and cycling durability.

Although the advantages of multi-dimensional conductive additives have been preliminarily validated in systems such as lithium iron phosphate [19], their application in high-nickel NCA cathodes remains limited, particularly lacking systematic evaluation under low-temperature conditions. Furthermore, existing studies predominantly focus on the impact of conductive additives on initial electrochemical performance, while understanding of conductive network evolution during cycling and its correlation with NCA structural degradation mechanisms remains insufficient.

Based on this context, the present work employs high-nickel NCA (LiNi_0.8_Co_0.15_Al_0.05_O_2_) as the cathode material to systematically compare the electrochemical performance of MWCNT/SP binary and GNPs/MWCNT/SP ternary conductive systems in 18650 cylindrical batteries. Through film resistivity measurements, standard charge–discharge tests, rate capability evaluation, low/high-temperature discharge characterization, electrochemical impedance spectroscopy analysis, and extended cycling tests, we elucidate the influence of GNP incorporation on electrode conductive networks, kinetic behavior, and cycling stability. The results demonstrate that the ternary conductive system significantly enhances battery cycling lifespan while maintaining excellent rate capability and low-temperature adaptability, providing experimental evidence and theoretical guidance for developing high-reliability low-temperature 18650 lithium-ion batteries.

## 2. Materials and Methods

### 2.1. Electrode Preparation and Cell Assembly

**Cathode Fabrication:** LiNi_0.8_Co_0.15_Al_0.05_O_2_ (NCA, D_50_ = 10–12 μm, specific surface area 0.8–1.2 m^2^/g, Tianjin B&M Science and Technology Co., Ltd., Tianjin, China) was employed as the cathode active material. The other raw materials include polyvinylidene fluoride (PVDF, Solef 5130, Solvay S.A., Brussels, Belgium) as the binder for all cathodes; Super carbon black (SP, Super C65, diameter ~60 nm, specific surface area 50 m^2^/g, Imerys Graphite & Carbon, Bodio, Switzerland) as the basic conductive agent; and multi-walled carbon nanotubes (MWCNT, diameter 10–20 nm, length 5–15 μm, purity >95%, Shenzhen Nanotech Port Co., Ltd., Shenzhen, China) and graphene nanoplatelets (GNP, XFNANO Materials Tech Co., Ltd., Nanjing, China) as the auxiliary conductive agents. Three conductive formulations were systematically investigated while maintaining constant total conductive additive loading (2.5 wt%) and active material content (96 wt%): (i) SP-only baseline: NCA:PVDF:SP = 96:1.5:2.5 (wt%); (ii) binary MWCNT/SP system: NCA:PVDF:SP:MWCNT = 96:1.5:1.5:1.0 (wt%), where MWCNT partially substituted SP; (iii) ternary GNP/MWCNT/SP system: NCA:PVDF:SP:MWCNT:GNP = 96:1.5:1.0:1.0:0.5 (wt%), incorporating GNP as the dimensional complement to MWCNT. Slurries were prepared by dispersing components in N-methyl-2-pyrrolidone (NMP) via planetary mixing, subsequently coated onto 15 μm aluminum foil, dried at 120 °C under vacuum (<100 Pa) for 12 h, calendered to a compaction density of 3.5 g/cm^3^, and punched into discs (*ϕ* = 14 mm for coin cells, *ϕ* = 65 mm for 18650 cylindrical cells) and further dried at 80 °C under vacuum for 24 h prior to cell assembly.

**Anode Fabrication:** Artificial graphite (GS-18, D_50_ = 10–20 μm, specific surface area 1.5–3.0 m^2^/g, BTR Co., Ltd., Shenzhen, China) was used as the anode active material, formulated as graphite:PVDF:SP = 95:3.5:1.5 (wt%). The slurry was coated onto 8 μm copper foil, dried, calendered, and processed following identical protocols to the cathodes.

**Cell Assembly:** Coin-type half-cells (CR2032) were assembled in an argon-filled glovebox (H_2_O and O_2_ < 0.1 ppm) with lithium metal foil as the counter electrode, Celgard 2400 separator, and 1.0 M LiPF_6_ in ethylene carbonate/dimethyl carbonate/ethyl methyl carbonate at 3:3:4 volume ratio with 5 wt% vinylene carbonate additive as the electrolyte. The 18650 cylindrical full cells were fabricated with graphite anodes at a negative-to-positive capacity ratio of 1.1:1. After electrode winding, cells were injected with 5.5 g electrolyte, sealed, and subjected to formation cycling (0.1C, 3 cycles) followed by aging at 45 °C for 48 h.

### 2.2. Cathode Resistivity and Microscopic Structure Characterization

Electrical volume resistivity was measured on calendered cathode films using the four-probe collinear method to eliminate contact resistance contributions at temperatures ranging from −40 °C to 50 °C with 10 °C intervals. The measurement configuration consisted of four equally spaced (1 mm) tungsten probes arranged linearly; a constant current (*I* = 1 mA) was applied through the outer probes, and the potential drop (*V*) was measured between the inner probes. Volume resistivity was calculated according to *ρ* = 2π*hV*/*I*, where *h* is the post-calendering film thickness measured by a micrometer caliper at five locations across each sample (edge, center, and three intermediate positions). At each temperature point, the thermal equilibrium criterion was defined as a sample temperature drift <0.1 °C over 5 min.

Electrode film samples (30 mm × 30 mm) were punched from coated aluminum foil and placed between two polished stainless steel plates with a constant uniaxial pressure of 5 MPa applied to ensure intimate electrical contact. Temperature control was achieved using a programmable chamber (ESPEC SH-242, accuracy: ±0.5 °C, ESPEC Corp., Osaka, Japan); samples were equilibrated at each set point for 30 min prior to measurement. Reproducibility was assessed through a multi-level sampling protocol: five electrode discs were prepared from independent coating batches for each formulation; each disc was measured at three distinct locations (center, edge, and intermediate); each location was measured three times. The reported *ρ* values represent the mean ± standard deviation (SD) of 45 total measurements (5 batches × 3 discs × 3 locations), unless otherwise noted. Batch-to-batch relative standard deviation was <5% for all formulations, confirming coating process reproducibility.

Morphological analysis on the composite microstructure of cathode film was performed using field-emission scanning electron microscopy (FE-SEM, Hitachi SU8010, Hitachi High-Tech Corp., Tokyo, Japan) at 5kV accelerating voltage. Cross-sectional samples were prepared by ion milling (Hitachi IM4000, Hitachi High-Tech Corp., Tokyo, Japan) to minimize mechanical damage.

Nitrogen adsorption–desorption isotherms were measured at 77 K using a Micromeritics ASAP 2460 analyzer (Micromeritics Instrument Corp., Norcross, GA, USA). Samples were degassed at 150 °C under vacuum for 12 h prior to measurement. The specific surface area was calculated using Brunauer–Emmett–Teller (BET) method in the relative pressure range *P*/*P*_0_ = 0.05–0.30. Pore size distribution and total pore volume were determined by the BJH (Barrett–Joyner–Halenda) method from the desorption branch. All measurements were performed on cathode film samples (30 mm × 30 mm) prepared under identical conditions to those used for electrochemical testing.

### 2.3. Electrochemical Characterization

**Standard Cycling and Rate Capability:** All electrochemical tests were conducted at 25 ± 1 °C unless otherwise specified. Cells were subjected to a standard formation protocol: constant-current (CC) charging at 0.2C (0.5 A) to 4.2 V, followed by constant-voltage (CV) holding at 4.2 V until the current decayed to 0.1 A (equivalent to 0.04C). Discharge was performed at various C-rates (0.2C, 3C, 7C, and 10C, where 1C = 2.5 A) with cutoff voltages of 2.8 V for 0.2C and 2.5 V for higher rates to accommodate increased polarization at elevated currents.

**Calorimetric Measurements:** The specific heat capacity of 18650 cells was determined using an accelerating rate calorimeter (ARC, THT Company, Milton Keynes, UK) under adiabatic conditions. Cells were subjected to controlled heating, and the temperature rise (Δ*T*) was recorded as a function of time (*t*) at constant heating power (*P*_1_). The specific heat capacity (*C*_h_) was calculated as *C*_h_ = *Q*/*m*∆*T* = *P*_1_*t*/*m*∆*T* = *P*_1_/*mσ* where *Q* denotes the total heat input, *m* is the cell mass, and *σ* = Δ*T*/*t* represents the heating rate (temperature–time slope).

**Thermal Behavior During Discharge:** After standard charging, cells were discharged at 10C (25 A) to 2.5 V. Voltage and surface temperature were recorded using a Keysight 34970A data acquisition system (sampling frequency: 1 Hz). The average heat generation rate (*Q*_m_) and instantaneous thermal power (d*Q*/d*t*) were derived from *Q*_m_ = *cm*∆*T* and d*Q/*d*t* = *cm*d*T*/d*t*, respectively.

**Pulse Discharge Characterization:** To assess extreme rate capability, a pulse discharge protocol was employed: CC discharge at 20C (50 A) for 200 ms, followed by a 1 h relaxation at 0.1C (0.25 A) to restore thermal and electrochemical equilibrium. This sequence was iterated until the cell voltage dropped below 2.5 V, enabling evaluation of pulse performance across the full state-of-charge (SOC) range.

**Low/High-Temperature Discharge:** Temperature-dependent electrochemical behavior was evaluated at −40 °C, 0 °C, and 50 °C using a programmable temperature chamber (BTH-150, Guangzhou Biaoji Instruments Co., Ltd., Guangzhou, China). Cells were thermally equilibrated at each set temperature for 2 h prior to testing to ensure uniform internal temperature distribution. Standard CC-CV charging (0.2C to 4.2 V, CV to 0.1 A) was conducted at 25 °C, followed by temperature equilibration and subsequent 1C discharge to 2.5 V at the target temperature.

**Electrochemical Impedance Spectroscopy (EIS):** EIS measurements were performed on a Bio-Logic VMP-300 potentiostat (Bio-Logic Science Instruments SAS, Seyssinet-Pariset, France) at open circuit potential, spanning a frequency range of 1 MHz to 10 mHz with an AC perturbation amplitude of 10 mV. Impedance spectra were fitted using ZView software (ZView4 3.5.0.10, Scribner Associates Inc., Southern Pines, NC, USA) with an appropriate equivalent circuit model to deconvolute ohmic resistance, charge-transfer resistance, and Warburg diffusion components.

**Self-Discharge and Capacity Recovery:** Charge retention characteristics were assessed by storing fully charged cells (100% SOC) at 25 °C for 30 days. Residual capacity was determined by 1C discharge to 2.5 V, with SOC retention calculated as the ratio of residual to initial capacity. A standard charge–discharge cycle was subsequently performed to evaluate capacity recovery rate and quantify irreversible capacity loss.

**Extended Cycling Stability:** Calendar aging was evaluated at 1C CC-CV charge/2C CC discharge for 1000 continuous cycles at 25 °C. Capacity retention was defined as (*C*_n_/*C*_max_) × 100%, where *C*_n_ is the discharge capacity of the nth cycle and *C*_max_ is the maximum discharge capacity observed during the cycling protocol.

## 3. Results and Discussion

### 3.1. Electrical Resistivity of Cathode Film

**Figure 1:**
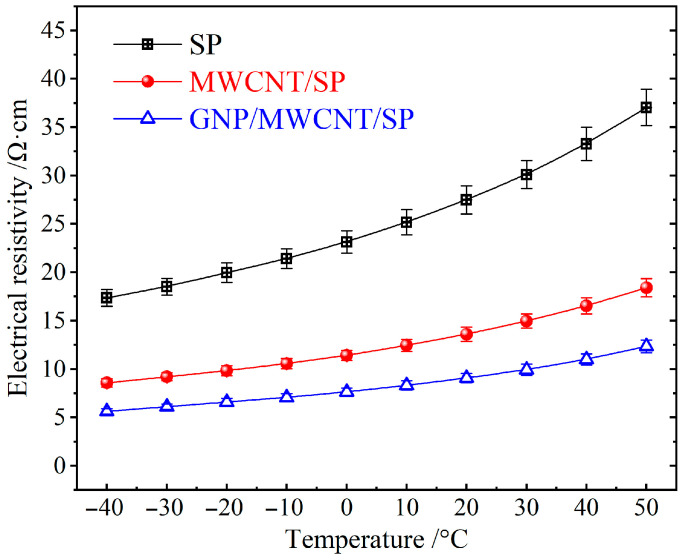
Temperature-dependent electrical resistivity of calendered NCA cathode films measured by the four-probe collinear method under 5 MPa uniaxial pressure. Error bars represent standard deviation from 45 measurements (5 independent coating batches × 3 locations × 3 repetitions).

Electrical resistivity of NCA cathode films with different conductive formulations was systematically investigated as a function of temperature, as shown in Figure 1. All electrodes exhibit a characteristic metallic-like behavior, with resistivity increasing monotonically as temperature decreased upon cooling from 50 °C to −40 °C. This behavior reflects the dominant contribution of the carbon-based conductive additives (GNP, MWCNT, SP), which exhibit metallic-like electron transport properties, rather than the semiconducting NCA active material.

All three conductive formulations maintain metallic-like conduction (positive temperature coefficient of resistivity) across the entire temperature range, confirming that 2.5 wt% loading exceeds the percolation threshold for each system. The MWCNT/SP binary system achieves a modest 49% reduction in resistivity at 20 °C compared to SP only (13.6 ± 0.7 vs. 27.5 ± 1.4 Ω·cm), while the GNP/MWCNT/SP ternary system demonstrates a more substantial 67% reduction (9.1 ± 0.5 Ω·cm). This difference highlights the critical role of 2D GNP in enhancing network connectivity beyond what 1D MWCNTs alone can achieve. Temperature sensitivity of electrical resistivity from −40 °C to 50 °C is comparable across all systems, increasing by 113% for SP, 115% for MWCNT/SP, and 119% for GNP/MWCNT/SP. This similarity indicates that once a continuous conductive network is established, the fundamental electron–phonon scattering mechanism dominates, with network topology having limited influence on temperature dependence.

It is significant considering that only 0.5 wt% GNP causes the synergistic effect derived from the complementary dimensional characteristics: GNPs provide extensive face-to-point contact coverage on NCA particle surfaces, while MWCNTs offer long-range line-to-point connections, and SP fills the residual interstitial voids. This hierarchical “point–line–plane” conductive architecture minimizes the percolation threshold and maximizes electrical transport efficiency. The 67% resistivity reduction achieved by the ternary system (from 27.5 ± 1.4 to 9.1 ± 0.5 Ω·cm at 20 °C) was reproducible across three independent coating batches (n = 45 total measurements per formulation), with a batch-to-batch relative standard deviation of ~5%. This statistical robustness confirms that the observed synergistic effect is intrinsic to the ternary formulation rather than an artifact of sample preparation variability. Nevertheless, the significant resistivity reduction achieved by the ternary system is more modest than the order-of-magnitude improvements sometimes reported in idealized systems [20,21]. This discrepancy can be attributed to several practical factors inherent in battery electrode fabrication as shown in Table 1. The high NCA active material loading (96 wt%) and required porosity for electrolyte infiltration create a tortuous, constrained environment where conductive network formation is inherently more difficult than in polymer–matrix composites typically studied in the literature [22].

It is noteworthy that the total conductive additive content remained constant at 2.5 wt% across all formulations, with nanocarbons substituting rather than supplementing SP. This experimental design isolates the structural effect of conductive network architecture from the dilution effect of additive loading, conclusively demonstrating that dimensional optimization of conductive additives, rather than mere quantity increase, governs the electrical transport properties of high-nickel cathode electrodes. In this context, a 50% resistivity reduction at constant 2.5 wt% loading represents a meaningful optimization, particularly given the additional benefits of improved mechanical stability and cycling durability.

### 3.2. Cathode Film Micromorphology

**Figure 2:**
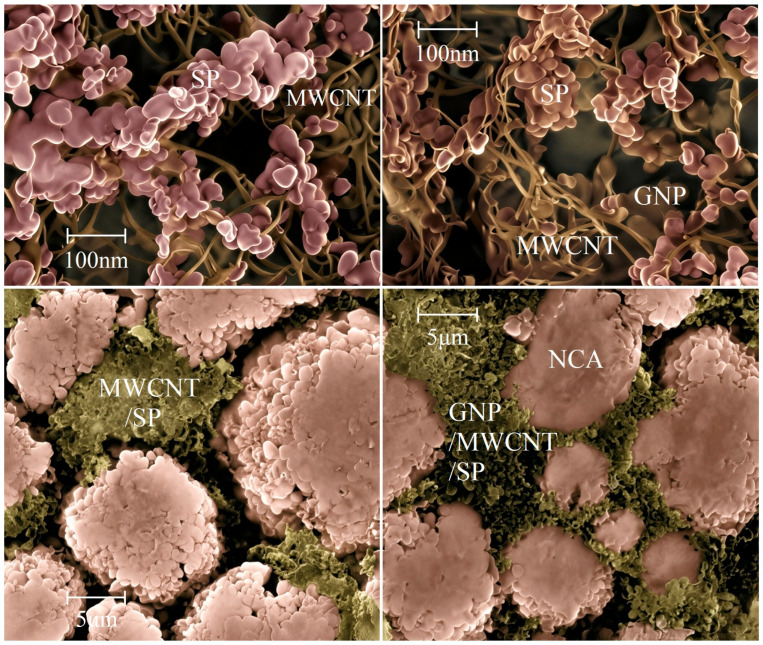
Cross-sectional SEM images of pristine conductive additives (**upper panels**) and corresponding cathode films (**lower panels**) in 18650 cells: MWCNT/SP binary system (**left panels**) and GNP/MWCNT/SP ternary system (**right panels**).

To elucidate the dispersion uniformity and network architecture of GNP/MWCNT/SP within the composite electrode, cross-sectional SEM imaging was performed on cathode films incorporating MWCNT/SP and GNP/MWCNT/SP conductive formulations, as shown in Figure 2. The MWCNTs exhibit a curved tubular morphology with diameters of approximately 10 nm, while the granular SP particles (~60 nm) interlace with the nanotubes, forming an interconnected three-dimensional conductive scaffold that bridges adjacent NCA active particles (Figure 2 left panels). However, the inherently high aspect ratio and entangled configuration of MWCNTs result in non-uniform dispersion, leaving discernible interfacial gaps between the conductive network and NCA particle surfaces in the binary MWCNT/SP electrode (Figure 2 left lower panel).

In contrast, the incorporation of GNPs fundamentally transforms the network topology. The two-dimensional graphene nanosheets intercalate extensively between MWCNTs and SP particles, establishing continuous conductive junctions that eliminate interstitial voids (Figure 2 right upper panel). This hierarchical integration enables more intimate and uniform interfacial contact between the ternary conductive architecture and NCA active material in the GNP/MWCNT/SP electrode (Figure 2 right lower panel). The complementary dimensional characteristics, where GNPs provide planar face-to-point coverage while MWCNTs maintain linear line-to-point bridging, collectively construct a mechanically robust and electronically percolated network, consistent with the substantially reduced resistivity measurements (Section 3.1).

The mechanistic advantage of the hierarchical architecture can be understood through a percolation theory framework: SP nanoparticles (0D) provide point contacts at local interstices, MWCNTs (1D) establish long-range linear conductive highways, and GNPs (2D) offer planar coverage that buffers mechanical stress and maintains interfacial integrity. This dimensional complementarity creates redundant electron transport pathways while suppressing the aggregation of any single component through steric hindrance effects.

### 3.3. Rate Capability and Thermal Behavior

**Figure 3:**
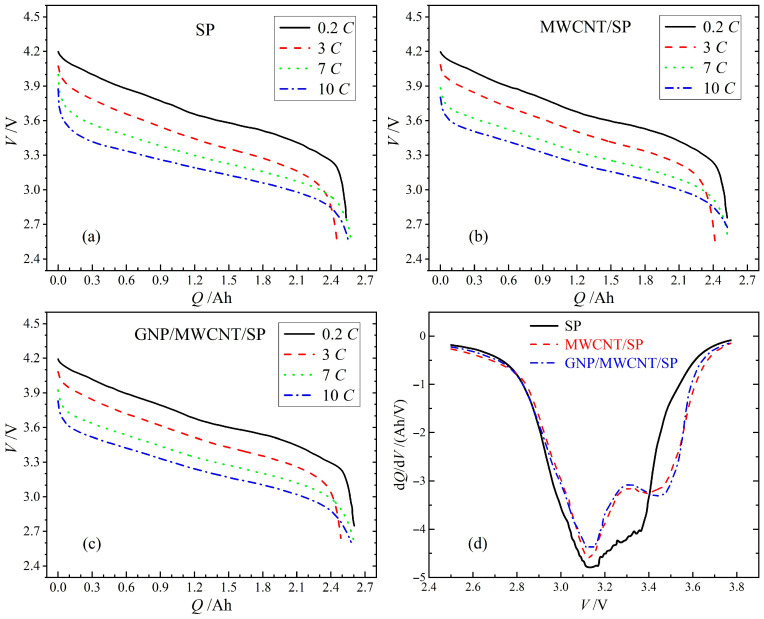
Electrochemical performance at various discharge rates: (**a**–**c**) galvanostatic discharge curves for SP-only, MWCNT/SP, and GNP/MWCNT/SP cells at 0.2C, 3C, 7C, and 10C; (**d**) corresponding d*Q*/d*V* curves highlighting polarization differences at 10C.

Rate capability was systematically evaluated at 0.2C, 3C, 7C, and 10C (1C = 2.5 A) to elucidate the influence of conductive additive dimensionality on high-power performance and thermal characteristics. All three 18650 configurations exhibit exceptional rate capability, with 10C discharge capacities of 2.56 Ah (SP-only), 2.58 Ah (MWCNT/SP), and 2.62 Ah (GNP/MWCNT/SP), corresponding to 101.3%, 101.8%, and 102.4% of their respective 0.2C capacities, as shown in Figure 3a–c. This marginal capacity increase with escalating current suggests minimal kinetic limitations, attributable to robust electronic networks facilitating efficient charge extraction even at elevated rates.

Differential capacity (d*Q*/d*V*) curves reveal distinct polarization signatures among the three conductive formulations, as indicated in Figure 3d. The SP-only cell exhibits a pronounced discharge peak centered at 3.40 V, whereas both nanocarbon-modified cells demonstrate peak potentials elevated above 3.50 V. Notably, the ternary GNP/MWCNT/SP system achieves the highest peak potential (3.52 V), marginally surpassing the binary MWCNT/SP system (3.51 V). The higher peak voltage indicating reduced polarization and superior rate performance correlates directly with the hierarchical conductive architecture: the 2D/1D composite network establishes continuous long-range electron transport pathways that maintain effective electrical contact across polydisperse active particles (D_50_ ≈ 10 μm), whereas the 0D SP alone provides only short-range point-to-point connections insufficient for high-rate operation.

The comparable performance enhancement observed for both binary and ternary nanocarbon systems suggests that the introduction of low-dimensional conductors, regardless of specific dimensionality, fundamentally transforms the electrode from diffusion-limited to electronically limited kinetics. The marginal capacity gains observed at 10C discharge (2.62 Ah for ternary vs. 2.58 Ah for binary, corresponding to a statistically insignificant difference of <2%) suggest that under ultra-high-rate operation, the rate-limiting step transitions from interparticle electron transport to solid-state lithium-ion diffusion within NCA active material. This interpretation is consistent with the electrochemical impedance spectroscopy analysis (Section 3.5), which reveals comparable Warburg coefficients and apparent lithium-ion diffusion coefficients (~9.4 × 10^−12^ cm^2^/s) across all three conductive formulations. The intrinsic solid-state diffusion coefficient of NCA (~10^−12^ to 10^−11^ cm^2^/s at 25 °C) is substantially slower than that of graphite anodes and constitutes the fundamental kinetic bottleneck in full-cell operation, rendering conductive additive optimization insufficient for further enhancing high-rate capacity beyond the binary system level.

To quantify thermal behavior during high-rate discharge, the specific heat capacity of 18650 cells was determined via adiabatic calorimetry. Given identical cell chemistry, design, and capacity with conductive additives constituting merely 0.78% of total cell mass, the specific heat capacity was assumed equivalent across all configurations. Six cells (two per formulation) were thermally coupled and subjected to controlled heating. At a heating rate *H*_r_ = 2 × 10^−3^ °C/s with applied power *P*_1_ = 0.62 W (3.25 V × 0.19 A) and total mass *m* = 0.27 kg, the specific heat capacity was calculated as *P*_1_/(*mH*_r_) = 1148 J/(kg·°C) which was subsequently employed for thermal power calculations during discharge operations.

**Figure 4:**
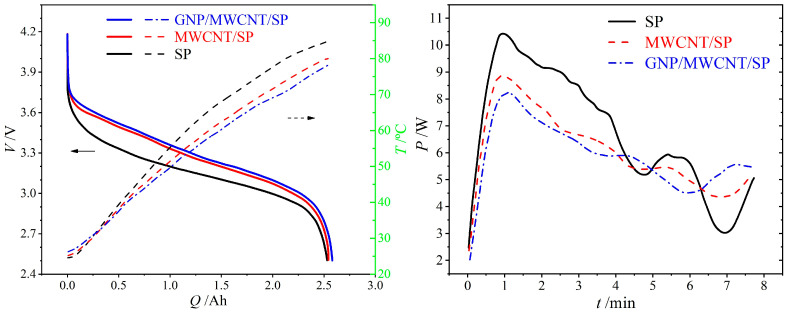
Temperature rise profiles (**left panel**) and instantaneous heat generation power (**right panel**) for cells with SP-only, MWCNT/SP, and GNP/MWCNT/SP conductive formulations.

Thermal profiles of 18650 cells subjected to continuous 20A (8C) discharge reveal pronounced distinctions among the three conductive formulations (Figure 4 left panel). The SP-only cell exhibits the lowest discharge voltage plateau and the highest temperature rise, reaching 60.3 °C. Incorporation of MWCNT or GNP/MWCNT conductive additives elevates the voltage plateau by approximately 0.2 V and reduces the final temperature by ~5 °C, attributable to the enhanced electronic conductivity and thermal dissipation capability of low-dimensional nanocarbons that mitigate electrochemical polarization and facilitate heat release. Notably, the ternary GNP/MWCNT/SP system achieves a marginally higher discharge plateau and lower terminal temperature (52.0 °C) compared to the binary MWCNT/SP system (54.8 °C), consistent with the superior electronic transport properties and reduced internal resistance of the hierarchical conductive architecture.

The instantaneous thermal power profiles (Figure 4 right panel) exhibit a characteristic rapid increase to a peak value at discharge initiation, followed by gradual decay. The SP-only cell reaches a peak heat generation rate of 10.4 W, whereas the MWCNT/SP and GNP/MWCNT/SP cells demonstrate substantially reduced peaks of 8.8 W and 8.2 W, respectively. This trend correlates directly with the electrode bulk resistivity measurements (Section 3.1), confirming that ohmic resistance dominates the thermal behavior during the initial discharge stage.

**Figure 5:**
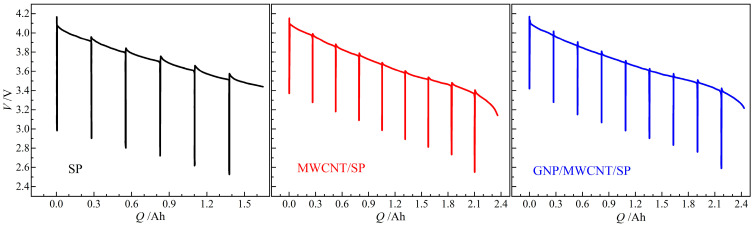
Pulse discharge capability at 20C (200 ms pulses) as a function of state-of-charge for the three conductive systems.

High-rate pulse performance was evaluated using 20C (50 A) discharge pulses (200 ms duration) interspersed at 10% SOC intervals to assess rate capability across the operational window, as shown in Figure 5. The SP-only cell sustains 20C pulses only above 50% SOC, with minimum voltage declining from 2.95 V at 100% SOC to 2.51 V at 50% SOC. In marked contrast, both nanocarbon-modified cells maintain 20C pulse capability down to 20% SOC. The MWCNT/SP and GNP/MWCNT/SP cells exhibit superior voltage retention, with minimum pulse voltages of 3.37 V and 3.42 V at 100% SOC, respectively, decreasing to 2.56 V and 2.59 V at 20% SOC. This enhanced low-SOC pulse performance underscores the critical role of robust electronic networks in sustaining high-rate operation under thermodynamically demanding conditions.

### 3.4. Temperature-Dependent Discharge Performance

**Figure 6:**
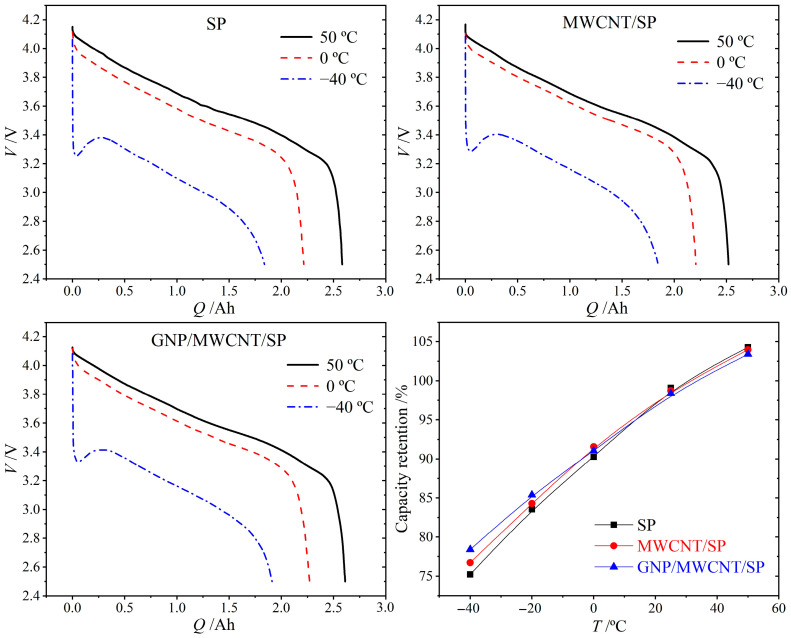
Temperature-dependent discharge curves at 1C for SP-only, MWCNT/SP, and GNP/MWCNT/SP conductive formulations.

The discharge characteristics of 18650 cells were evaluated at 50 °C, 0 °C, and −40 °C (1C rate) to assess the influence of conductive additive dimensionality on extreme-temperature operability, as illustrated in Figure 6. All three formulations exhibit comparable capacity retention across the tested temperature range. At −40 °C, the cells achieve retention values of 75.2% (SP-only), 76.3% (MWCNT/SP), and 76.8% (GNP/MWCNT/SP) relative to 25 °C capacity. The marginal improvement (<1%) conferred by the ternary GNP/MWCNT/SP system over the binary MWCNT/SP counterpart suggests that conductive additive optimization alone yields diminishing returns for low-temperature performance. This observation reflects the competing effects of electronic conductivity and internal heating: reduced ohmic resistance mitigates low-temperature polarization, yet simultaneously diminishes resistive heating that would otherwise elevate internal temperature and enhance discharge kinetics [23].

**Figure 7:**
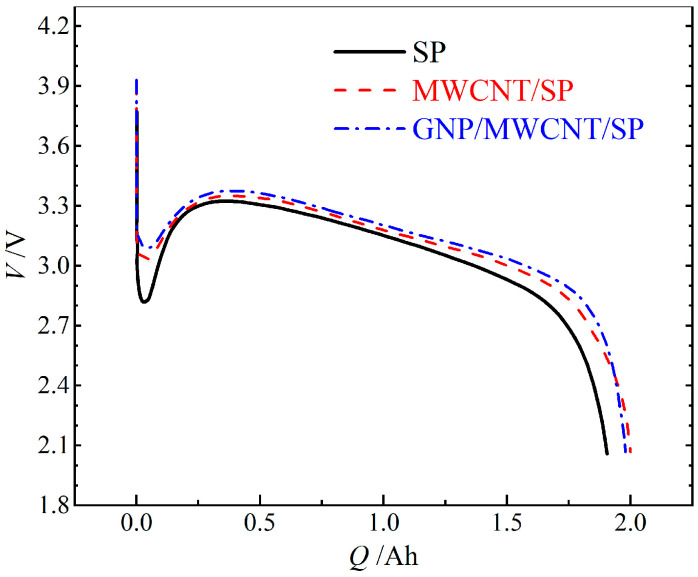
Discharge voltage profiles at −40 °C/2C, illustrating the initial voltage drop and subsequent thermal recovery behavior for the three conductive systems.

To elucidate the interplay among polarization, ohmic heating, and discharge capacity under demanding conditions, cells were subjected to −40 °C/2C discharge, as shown in Figure 7. Despite identical initial temperatures, the three formulations exhibit distinct voltage responses. The SP-only cell demonstrates the most severe initial voltage drop to ~2.8 V, consistent with its highest electrode resistivity. Both the nanocarbon modified cells show substantially reduced polarization: the MWCNT/SP cell maintains >2.9 V, while the GNP/MWCNT/SP cell achieves the smallest voltage drop (>3.0 V), confirming the superior electronic transport efficiency of the hierarchical conductive network under high-rate, low-temperature conditions.

As discharge progresses, substantial ohmic heat generation elevates internal temperature and attenuates polarization, causing voltage recovery to ~3.3 V in all cells. Notably, the SP-only cell exhibits the most pronounced voltage rebound due to its highest resistive heating, yet ultimately delivers the lowest discharge capacity as elevated internal resistance reasserts dominance upon thermal equilibration. These results demonstrate that while conductive additive engineering effectively reduces initial polarization penalties, the fundamental limitation on low-temperature performance resides in the activated solid-state lithium-ion diffusion within NCA active material, whose diffusion coefficient decreases by orders of magnitude upon cooling, overwhelming the incremental gains from enhanced electronic conductivity.

The comparable low-temperature capacity retention across all formulations (75.2% for SP-only, 76.3% for MWCNT/SP, and 76.8% for GNP/MWCNT/SP at −40 °C, with <2% absolute variation) indicates that at extreme subzero temperatures, the drastically reduced solid-state lithium-ion diffusion coefficient within NCA active material—decreasing by approximately two orders of magnitude upon cooling from 25 °C to −40 °C—becomes the dominant performance bottleneck, overwhelming the incremental gains from enhanced electronic conductivity. This explains why ternary optimization yields diminishing returns for low-temperature discharge capacity, whereas its primary advantage manifests in extended cycling stability where interfacial mechanical degradation and contact loss, rather than ionic transport, dominate capacity fade. The competing effects of electronic conductivity and internal heating further complicate this picture: reduced ohmic resistance mitigates low-temperature polarization yet simultaneously diminishes resistive self-heating that would otherwise elevate internal temperature and transiently enhance discharge kinetics.

Thermogravimetric–mass spectrometry (TG-MS) analysis is proposed to further substantiate the temperature–performance correlation, and preliminary expectations based on the pore structure analysis suggest the following. (i) Low-temperature gas evolution: at −40 °C, the SP-only electrode, with its micropore-dominated structure (most probable pore: 8.5 nm), would exhibit restricted electrolyte infiltration and enhanced polarization, leading to localized Li plating and subsequent decomposition (CO_2_, *m*/*z* = 44; CH_4_, *m*/*z* = 16); in contrast, the mesoporous architecture (15.8 nm) of the GNP/MWCNT/SP system ensures uniform electrolyte distribution, thereby suppressing gas evolution. (ii) Thermal runaway precursors: during heating (25 → 300 °C), TG-MS would detect differential gas release profiles, and the GNP/MWCNT/SP system is anticipated to show a delayed onset of CO_2_ evolution—indicating suppressed electrolyte oxidation—due to the protective face-to-point coverage of GNP nanosheets that physically isolate NCA particles from direct electrolyte contact. (iii) Correlation with EIS: the charge-transfer resistance trend at low temperature (SP > MWCNT/SP > GNP/MWCNT/SP, Table 2 in Section 3.5) should inversely correlate with the intensity of gas evolution peaks, confirming that enhanced pore connectivity mitigates polarization-driven side reactions.

### 3.5. Electrochemical Impedance Spectroscopy

**Figure 8:**
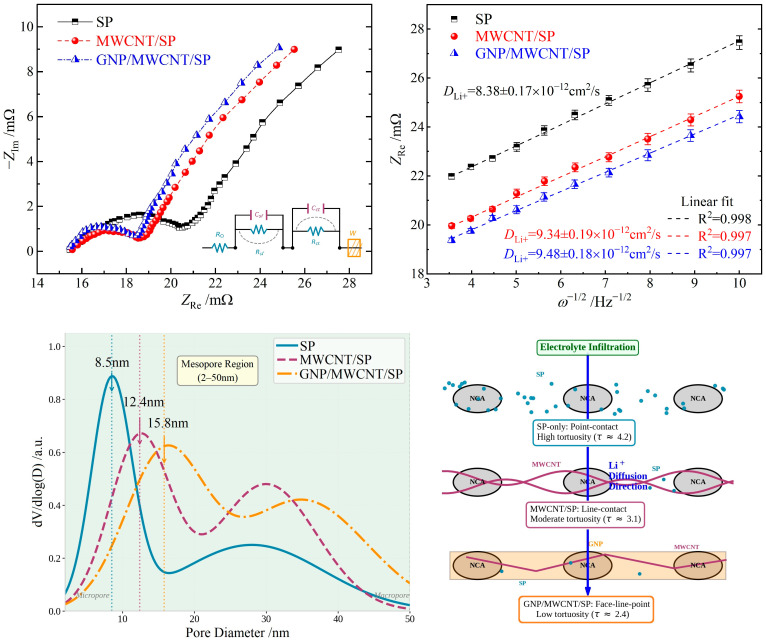
Nyquist plots for 18650 cells with different conductive formulations (**upper-left panel**) with an inset equivalent circuit diagram and linear fitting of *Z*_Re_ versus *ω*^−1/2^ in low-frequency region for *D*_Li_^+^ calculation (**upper-right panel**) with error bars (SD) from three independent measurements; BET analysis of NCA cathode films with different conductive additive formulations: pore size distribution curves derived from BJH method (**lower-left panel**) and schematic illustration of pore architecture and Li^+^ diffusion pathways (**lower-right panel**).

Electrochemical impedance spectroscopy was employed to deconvolute the kinetic contributions of distinct electrode processes. The Nyquist spectra for all three 18650 configurations display a high-frequency intercept corresponding to ohmic resistance (*R*_Ω_), followed by one or two partially overlapped semicircles in the high-to-medium-frequency region attributed to surface film resistance (*R*_sf_) and charge-transfer resistance (*R*_ct_), respectively, and a low-frequency linear tail representing Warburg impedance associated with solid-state lithium-ion diffusion (Figure 8 upper-left panel). Equivalent circuit fitting yields the impedance parameters summarized in Table 2. The SP-only cell exhibits the highest *R*_Ω_, *R*_sf_, and *R*_ct_ values, confirming that low-dimensional nanocarbon additives substantially enhance electrode kinetics by improving electronic connectivity and interfacial charge transfer. Both nanocarbon-modified cells demonstrate reduced impedance magnitudes. Notably, the ternary GNP/MWCNT/SP system achieves the lowest *R*_sf_ and *R*_ct_ due to the hierarchical conductive architecture that optimizes both ionic accessibility and electron transport across the electrode–electrolyte interface.

**Table 2:** Impedance parameters derived from EIS analysis using equivalent circuit fitting (mean ± SD, n = 3).

Cathode Conductive Agent	*R*_Ω_/mΩ	*R*_sf_/mΩ	*R*_ct_/mΩ	*σ*/10^−4^
SP	16.23 ± 0.42	5.20 ± 0.24	114.98 ± 2.35	8.36 ± 0.23
MWCNT/SP	15.95 ± 0.32	4.58 ± 0.18	75.79 ± 1.59	8.01 ± 0.21
GNP/MWCNT/SP	15.84 ± 0.26	4.36 ± 0.15	65.66 ± 1.48	7.92 ± 0.19

The low-frequency Warburg region was analyzed to estimate the apparent lithium-ion diffusion coefficient (*D*_Li+_) using the following relationships [24]:

*Z*_Re_ = *R*_s_ + *R*_ct_ + *σω*^−1/2^ (1)

*D*_Li+_ = R^2^*T*^2^/2*A*^2^*n*^4^*F*^4^*C*_Li+_^2^*σ*^2^ (2)

where R is the gas constant, *T* is absolute temperature (298.15 K), *A* is the active material surface area, *F* is Faraday’s constant, *n* is the number of transferred electrons, *ω* is angular frequency, *C*_Li+_ is Li^+^ concentration, and *σ* is the Warburg coefficient. The linear regression of *Z*_re_ versus *ω*^−1/2^ yields *D*_Li+_ shown in the upper-right panel in Figure 8.

The calculated *D*_Li_^+^ values are comparable across all three formulations, consistent with the understanding that solid-state lithium-ion diffusion within NCA active material (10^−12^~10^−9^ cm^2^/s) is substantially slower than that in graphite anodes (10^−10^~10^−9^ cm^2^/s) and thus constitutes the rate-limiting step in full-cell operation [25,26]. The negligible variation in *D*_Li_^+^ among the conductive systems confirms that low-dimensional additives primarily enhance interparticle electronic conductivity rather than intraparticle ionic transport, as their spatial distribution is confined to the conductive matrix rather than the active material bulk.

The BET analysis reveals that the ternary GNP/MWCNT/SP system achieves an optimal pore architecture: while its total porosity (30.8%) is comparable to the binary system (31.2%), the average pore diameter (22.6 nm vs. 18.3 nm) and pore volume (0.218 vs. 0.189 cm^3^/g) are substantially larger, as shown in the lower panels of Figure 8 and Table 3. This hierarchical pore structure arises from the intercalation of GNP nanosheets between MWCNT bundles, which eliminates micropores (<2 nm) that are inaccessible to solvated Li^+^ (effective diameter ~0.9–1.2 nm in the electrolyte) while creating well-connected mesoporous channels. The hierarchical conductive architecture not only enhances electronic conductivity (Section 3.1) but also optimizes the pore structure for ion transport. This mesopore-dominated structure reduces ionic tortuosity and ensures uniform electrolyte distribution, which is critical for low-temperature operation where sluggish Li^+^ diffusion constitutes the rate-limiting step (Section 3.4). The reduced tortuosity (*τ*_ion_) in the ternary system estimated from the Bruggeman relation *τ*_ion_ = *ε*^−1/2^, where *ε* is porosity, enhances the effective Li^+^ diffusion coefficient (*D*_eff_ = *D*_bulk_/*τ*_ion_), consistent with the EIS-derived Warburg coefficients. Furthermore, the lower BET surface area (38.6 m^2^/g) compared to SP only (48.5 m^2^/g) reduces parasitic reactions at the electrode–electrolyte interface, contributing to the improved cycling stability.

### 3.6. Charge Retention and Capacity Recovery

One-dimensional nanocarbon conductive additives frequently contain trace magnetic impurities that may catalyze parasitic reactions and exacerbate self-discharge. To quantify this effect, charge retention tests were conducted on fully charged cells stored at 25 °C for 30 days (Table 4). All cells employed low-temperature electrolyte formulations, resulting in marginally lower retention values compared to conventional systems optimized for ambient operation. The SP-only cell exhibits the highest charge retention (>93%) and near-complete capacity recovery (99%), indicating minimal parasitic activity. In contrast, both nanocarbon-modified cells show modestly reduced retention: 92.3% for MWCNT/SP and 92.8% for GNP/MWCNT/SP, with equivalent 99% recovery rates. The marginal deterioration (<1% absolute) confirms that magnetic impurities in MWCNTs exert a detectable yet limited influence on self-discharge under the tested conditions. Notably, the ternary system exhibits slightly superior retention relative to the binary counterpart, possibly attributable to the reduced MWCNT content (1.0 wt% vs. 1.5 wt%) and the stabilization of GNP surface passivation.

### 3.7. Extended Cycling Stability

**Figure 9:**
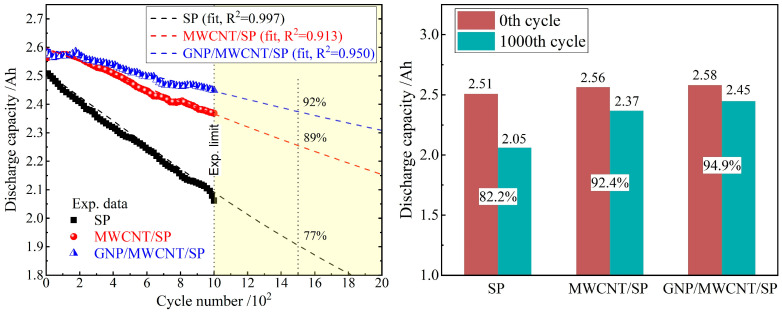
Cycling stability at 1C charge/2C discharge for 1000 cycles: discharge capacity versus cycle number (**left panel**) and capacity retention comparison for the three conductive formulations (**right panel**). The vertical dot lines indicate 1000 and 1500 cycles.

Low-dimensional nanocarbon conductive additives are widely recognized to enhance cycling durability through mechanical and electronic stabilization of composite electrodes. To elucidate the distinct contributions of GNP and MWCNT to long-term performance, 18650 cells were subjected to 1000 continuous cycles at 1C charge/2C discharge, as shown in Figure 9. While 1000 cycles provide statistically significant differentiation among conductive formulations and establish clear performance hierarchies, we acknowledge that this cycle number remains limited for full qualification of 18650 NCA systems in commercial applications, where lifespans exceeding 1500–2000 cycles are typically required. Accordingly, the term “long-cycle performance” should be interpreted as “substantially enhanced cycling durability over 1000 cycles” rather than an absolute claim of commercial long-life capability. Nevertheless, long-cycling stability can be approximately predicted by bi-exponential fitting and extrapolation on the discharge capacity decaying profile in 1000 cycles, as shown by the left panel of Figure 9.

The SP-only cell exhibits rapid capacity fade, declining from 2.51 Ah to 2.06 Ah (82.2% retention), consistent with the degradation of discrete point-to-point conductive contacts under mechanical stress and thermal fluctuations. In marked contrast, both nanocarbon-modified systems demonstrate substantially enhanced longevity. The MWCNT/SP cell achieves 92.4% retention (2.56 to 2.37 Ah), while the ternary GNP/MWCNT/SP system attains superior stability with 94.9% retention (2.58 to 2.45 Ah). This performance hierarchy of ternary > binary >> SP only confirms the critical role of dimensional hierarchy in sustaining electrode integrity over extended cycling.

To deconvolute the capacity fade mechanisms and project long-term behavior beyond the experimentally tested 1000 cycles, the discharge capacity versus cycle number was fitted to a bi-exponential decay model, *C*(*N*) = *C*_0_[A·exp(−*k*_1_·*N*) + B·exp(−*k*_2_·*N*)], where *C*_0_ is the initial capacity, *N* is the cycle number, *k*_1_ and *k*_2_ are the fast and slow decay rate constants, respectively, and A + B = 1. The fast decay component (*k*_1_) is attributed to interfacial instability and conductive network degradation, while the slow decay component (*k*_2_) reflects active material structural degradation. Fitting yields *k*_1_/*k*_2_ ratios of 5.5 (SP only) and 1.0 (MWCNT/SP and GNP/MWCNT/SP) with coefficients of determination R^2^ > 0.91 for all formulations. The substantially lower *k*_1_/*k*_2_ ratio for both the binary and ternary systems indicates that 1D–2D nanoconductives primarily suppress interfacial contact loss—the dominant degradation mode in the initial cycling period—rather than merely slowing intrinsic active material degradation. It is predicted by the bi-exponential decay model that the capacity retentions approach 77% (SP only), 89% (MWCNT/SP), and 92% (GNP/MWCNT/SP) at 1500 cycles, as indicated by the fitting curves in the left panel of Figure 9.

The marked improvement conferred by nanocarbon additives originates from their high aspect ratios and specific surface areas, which establish robust three-dimensional conductive scaffolds that accommodate cyclic volume changes in NCA active material. The electrochemical impedance analysis (Section 3.5) enables deconvolution of the individual contributions: MWCNT primarily preserves long-range electron pathways by maintaining tube-to-particle electrical contact, thereby preventing electronic isolation of fragmented active material; GNP predominantly maintains intimate interfacial contact and suppress particle surface rock-salt phase propagation through their conformal encapsulation layer. Therefore, the hierarchical GNP/MWCNT/SP architecture provides distinct mechanistic advantages: GNP nanosheets deliver planar face-to-point coverage that buffers particle expansion and maintains interfacial contact, while MWCNTs supply flexible line-to-point bridges that preserve long-range electron transport pathways. This synergistic stabilization mitigates electronic conductivity degradation and reduces resistive heating, which collectively suppress thermal–mechanical degradation and extend cycle life. The superior retention of the ternary system (94.9%) relative to the binary counterpart (92.4%), despite comparable initial resistivities and rate performances, underscores that this dimensional complementarity (2D + 1D) outperforms single-dimensional nanocarbon reinforcement (1D alone) in sustaining electrode structural integrity under prolonged electromechanical stress.

While the present study establishes the performance superiority of the ternary conductive system through electrochemical and morphological characterization, atomic-scale insights into the structure–performance correlation would benefit from advanced techniques. Operando XRD could elucidate how the hierarchical conductive network influences the lattice parameter evolution (particularly c-axis expansion) of NCA during delithiation, while HRTEM would enable direct visualization of surface reconstruction layer (rock-salt phase) thickness on cycled particles. Additionally, Raman spectroscopy (analyzing the D/G intensity ratio and 2D band features) could correlate the defect density and stress state of GNP/MWCNT with their electronic conductivity evolution upon cycling. These complementary analyses would strengthen the mechanistic understanding of how dimensional synergy suppresses mechanical degradation and maintains interfacial charge-transfer kinetics.

### 3.8. Quantitative Correlation Analysis of Conductive Network Robustness and Cycling Stability

**Figure 10:**
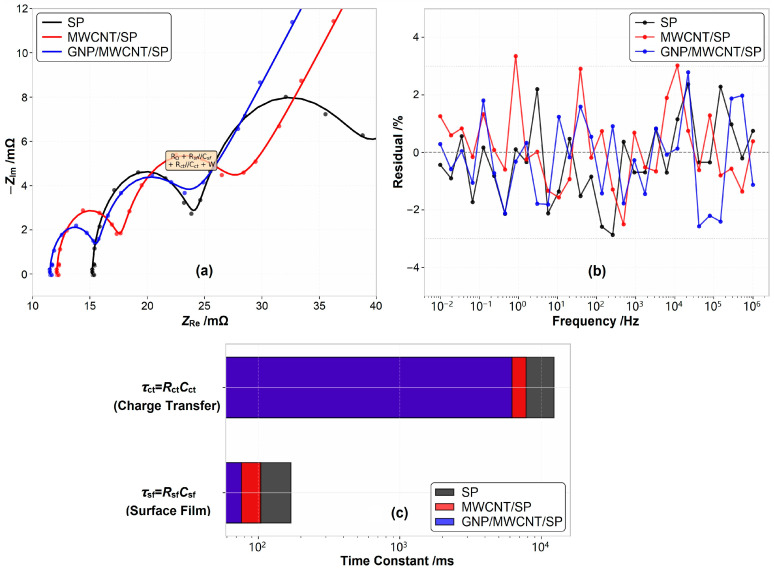
(**a**) Nyquist plots with fitted curves (solid lines) based on equivalent circuit model and experimental data (scattered points), (**b**) residual distribution confirming random fitting errors within ±3%, and (**c**) characteristic time constants *τ*_sf_ and *τ*_ct_ distinguishing surface film and charge-transfer processes.

The electrochemical impedance spectra presented in Figure 8 were subjected to rigorous equivalent circuit modeling to extract physically meaningful parameters and validate the quantitative reliability of the impedance analysis. Figure 10a presents the Nyquist plots with overlaid fitted curves (solid lines) based on the equivalent circuit model R_Ω_ + *R*_sf_//*C*_sf_ + *R*_ct_//*C*_ct_ + *W*, where *C*_sf_ and *C*_ct_ denote surface film capacitance and double-layer capacitance, and *W* represents Warburg impedance associated with solid-state lithium-ion diffusion. The goodness-of-fit was evaluated using the chi-squared (χ^2^) test and residual analysis. All three formulations yield χ^2^ values below 2.5 × 10^−3^, with coefficients of determination R^2^ > 0.998, confirming excellent agreement between experimental data and the proposed model. The residual distribution as a function of frequency demonstrates random scatter within ±3% across the entire frequency range (1 MHz to 10 mHz), as shown in Figure 10b. This random residual pattern in the absence of systematic bias validates that the equivalent circuit adequately captures the underlying electrochemical processes without overfitting or model misspecification.

The characteristic time constants *τ*_sf_ = *R*_sf_ × *C*_sf_ and *τ*_ct_ = *R*_ct_ × *C*_ct_ provide further mechanistic insight, as shown in Figure 10c. The charge-transfer time constant *τ*_ct_ decreases from 12.3 s (SP) to 6.2 s (ternary), reflecting accelerated electrochemical reaction kinetics at the active material interface. Conversely, the surface film time constant *τ*_sf_ decreases from 170 ms to 76 ms, indicating a thinner, more stable surface film. The temporal separation of *τ*_sf_ (milliseconds) versus *τ*_ct_ (seconds) enables independent optimization: the ternary system simultaneously accelerates charge transfer (beneficial for rate capability) and stabilizes the surface film (critical for long-term cycling), whereas binary MWCNT/SP optimization primarily affects the latter.

**Figure 11:**
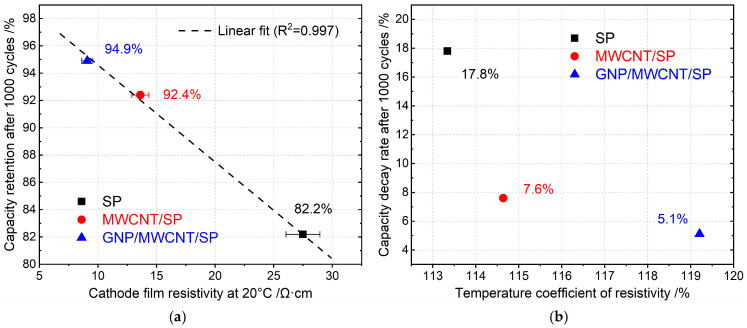
(**a**) Strong negative correlation between initial cathode resistivity and 1000-cycle capacity retention, establishing resistivity as a predictive metric for cycling stability, and (**b**) decoupling of temperature coefficient of resistivity (intrinsic property) from capacity decay rate (engineering performance).

To establish a quantitative and predictive relationship independent of post-cycling microstructural characterization, we analyzed the correlation between initial cathode film resistivity (measured at 20 °C, Section 3.1) and capacity retention after 1000 cycles (Section 3.7). Figure 11a presents this cross-parameter analysis, revealing a strong negative linear correlation (R^2^ = 0.997) between resistivity and cycling stability. The linear regression yields a slope of −0.52% retention per Ω·cm resistivity reduction, with the ternary system (9.1 ± 0.5 Ω·cm, 94.9% retention) occupying the optimal correlation. This near-unity correlation coefficient is statistically significant (*p* < 0.001) and physically meaningful, indicating that initial electrode resistivity as a direct measure of conductive network completeness represents a reliable predictor of extended cycling stability. The correlation implies that every 1 Ω·cm reduction in resistivity translates to approximately 0.5% improvement in capacity retention, providing an engineering design criterion for conductive additive optimization.

A critical mechanistic question is whether the observed cycling stability differences originate from intrinsic material properties (e.g., temperature-dependent electron transport) or engineered network topology. To address this, Figure 11b compares the temperature coefficients of resistivity (TCR defined as the relative resistivity change from −40 °C to 50 °C) against the capacity decay rate after 1000 cycles. The TCR values are remarkably similar across all formulations: 113% (SP), 115% (MWCNT/SP), and 119% (GNP/MWCNT/SP). This narrow range (±3%) indicates that once a continuous conductive network is established, the fundamental electron–phonon scattering mechanism, which governs temperature-dependent resistivity, is insensitive to network topology. The decoupling of TCR and decay rate provides decisive evidence that long-term cycling stability is governed by network mechanical robustness and interfacial stability (engineering parameters), rather than intrinsic electronic transport properties (material parameters). This distinction directly supports the central thesis of this work: dimensional optimization of conductive additives enhances cycling stability through topological and mechanical advantages (GNP encapsulation, MWCNT bridging, steric hindrance against aggregation), not through modification of the fundamental electronic conduction mechanism.

### 3.9. Future Perspectives

**Life Cycle Assessment (LCA) Perspective:** While the ternary GNP/MWCNT/SP system demonstrates superior electrochemical performance, the environmental and economic implications of nanocarbon additives warrant consideration. Preliminary analysis suggests that although GNP and MWCNT production involves higher energy consumption than conventional carbon black, the 15% improvement in cycle life (94.9% vs. 82.2% retention at 1000 cycles) significantly reduces the battery replacement frequency, potentially lowering the overall environmental impact per kWh delivered over the service life. A comprehensive LCA comparing cumulative energy demand and global warming potential across the three formulations will be conducted in future work.

**Computational Insights:** Density functional theory (DFT) calculations are proposed to elucidate the atomistic origins of the GNP stabilization effect. Specifically, adsorption energy calculations for GNP edge sites (zigzag vs. armchair configurations) on NCA (003) surfaces would quantify the interfacial binding strength, while nudged elastic band (NEB) method could determine Li^+^ diffusion barriers at the GNP–electrolyte interface. These computational results would complement the experimental observations by establishing structure–property relationships at the electronic level.

**Advanced Characterization Roadmap:** Future studies will incorporate operando XRD to monitor real-time lattice parameter evolution during low-temperature cycling, and operando Raman to track the stress/strain state of the carbon conductive network. These techniques will provide direct evidence for the protective role of the hierarchical architecture under extreme conditions. To further strengthen the structure–performance correlation with minimal additional experimental burden, we prioritize the following characterization sequence: (i) ex situ HRTEM of cycled electrodes to quantify surface reconstruction layer thickness; (ii) Raman mapping to correlate carbon defect density with local electrochemical activity; and (iii) operando XRD during low-temperature cycling to track lattice parameter evolution. These targeted analyses would provide definitive evidence for the mechanistic advantages proposed herein without requiring extensive instrumental resources.

## 4. Conclusions

A dimensionally complementary ternary conductive additive design (0D SP/1D MWCNT/2D GNP) decouples the conductivity–stability paradox in high-nickel NCA cathodes under low-temperature operation by establishing a multiscale percolation network with synergistic electron–ion transport pathways. The following mechanistic conclusions are drawn from the systematic experimental investigation:

(1) Hierarchical conductive architecture and electrical transport: By harnessing complementary “point–line–plane” dimensional synergy, where GNP nanosheets provide face-to-point coverage, MWCNTs offer line-to-point bridging, and SP ensures percolation through point-to-point contacts, and the ternary system reduces cathode resistivity by 67% (to 9.1 Ω·cm) compared to conventional SP. Cross-sectional SEM confirms that this hierarchical architecture eliminates interstitial voids and establishes intimate interfacial contact with NCA particles. Notably, this optimization is achieved at constant 2.5 wt% total conductive additive loading, demonstrating that dimensional optimization of conductive additives, rather than increasing quantity, governs the electrical transport properties of high-loading nickel-rich cathodes.

(2) Kinetic bottleneck identification under extreme conditions: The fabricated 2.5 Ah 18650 cylindrical battery achieves concurrent high-rate, low-temperature, and extended-cycling performance rarely realized in single systems: 76.8% capacity retention at −40 °C (1C), 10C continuous and 20C pulse discharge capability, and 94.9% capacity retention after 1000 cycles (1C/2C). However, electrochemical impedance spectroscopy reveals that the apparent Li^+^ diffusion coefficients (~9.4 × 10^−12^ cm^2^/s) are statistically indistinguishable across all conductive formulations, confirming that solid-state Li^+^ diffusion within NCA active material constitutes the intrinsic rate-limiting step. Consequently, while ternary optimization substantially reduces charge-transfer resistance and ohmic polarization, its impact on high-rate capacity and low-temperature discharge is bounded by ionic transport limitations, explaining the marginal improvements (<2%) relative to the binary system in these metrics.

(3) Degradation mechanism and dimensional synergy in cycling stability: The primary advantage of the ternary system manifests in long-term cycling stability, where the hierarchical network suppresses capacity fade to 5.1% over 1000 cycles compared to 7.6% (binary) and 17.8% (SP only). This superiority originates from the mechanistic complementarity of GNP and MWCNT: GNP nanosheets deliver planar face-to-point coverage that buffers particle expansion and maintains interfacial contact against mechanical stress, while MWCNTs supply flexible line-to-point bridges that preserve long-range electron transport pathways against conductive network fragmentation. The reduced resistive heating (peak thermal power: 8.2 W vs. 10.4 W for SP only) further mitigates thermally accelerated degradation. The divergence between cycling stability (substantially improved) and rate/low-temperature performance (marginally improved) establishes a critical design principle: conductive additive optimization maximally impacts electrode processes limited by electronic conductivity and interfacial stability, rather than those limited by active material intrinsic ionic diffusion.

## Figures and Tables

**Table 1:** Comparison of ideal vs. achieved resistivity reduction.

Factor	Ideal System	Practical Electrode	Impact (↓: Decrease; ↑: Increase)
Filler dispersion	Individual CNT/GNP	Aggregates and bundles	↓ Effective aspect ratio
Active material interference	Pure polymer matrix	NCA particles (90% mass)	↓ Network continuity
Binder distribution	Uniform coating	PVDF domains	↓ Conductive pathway isolation
Calendering compression	None	High pressure (5–10 MPa)	Densification, contact improvement
Porosity requirements	Minimal	~30% for electrolyte access	↑ Tortuosity, ↓ Connectivity

**Table 3:** BET surface area and pore structure parameters of NCA cathode films with different conductive formulations.

Conductive Formulation	Specific Surface Area/(m^2^/g)	Pore Volume/(cm^3^/g)	Average Pore Diameter/nm	Most Probable Pore Diameter/nm	Porosity /%	Tortuosity *τ*_ion_
SP-only	48.5 ± 2.1	0.152 ± 0.008	12.5 ± 0.8	8.5	28.5 ± 0.9	4.2 ± 0.3
MWCNT/SP	42.3 ± 1.8	0.189 ± 0.010	18.3 ± 1.1	12.4	31.2 ± 1.0	3.1 ± 0.2
GNP/MWCNT/SP	38.6 ± 1.5	0.218 ± 0.012	22.6 ± 1.3	15.8	30.8 ± 1.1	2.4 ± 0.2

**Table 4:** Charge retention and capacity recovery for 18650 batteries with different cathode conductive agents.

Cathode Conductive Agent	Initial Capacity/Ah	Retained Capacity/Ah	Capacity Retention/%	Recovered Capacity/Ah	Recovery Efficiency/%
SP	2.541	2.372	93.3	2.518	99.1
MWCNT/SP	2.564	2.356	91.9	2.525	98.5
GNP/MWCNT/SP	2.6065	2.417	92.7	2.579	98.9

## Data Availability

The original contributions presented in this study are included in the article. Further inquiries can be directed to the corresponding author.
